# Electronic cigarettes and vaping: toxicological awareness is increasing

**DOI:** 10.1007/s00204-020-02786-3

**Published:** 2020-05-21

**Authors:** Hermann M. Bolt

**Affiliations:** Department of Toxicology, Leibniz Research Centre for Working Environment and Human Factors at TU, Dortmund (IfADo), Ardeystr. 67, 44139 Dortmund, Germany

Almost 6 years ago, a Guest Editorial of Archives of Toxicology was published by Henkler and Luch (2014), pointing to toxicological problems associated with the use of e-cigarettes. From a regulatory point of view, the discussion was primarily focused on nicotine, as a proposal was just approved at the EU level to set an upper limit of 20 mg nicotine per ml vaping liquid. But at the same time, it was also known that vaping liquids contained a wide range of flavourings, additives and contaminants, a field that was insufficiently investigated (Hutzler et al. 2014).

Since that, e-cigarettes have received rapidly increasing popularity. The dynamics of this development is evidenced by very recent publications from all over the world (e.g., Shiffman and Sembower 2020 [USA]; Al Rifai et al. 2020 [USA]; Cruz-Jiménez et al. 2020 [Mexico]; Sharan et al. 2020 [India]; Kapan et al. 2020 [Europe]; Wipfli et al. 2020 [East Asia], Briganto et al. 2020 [world-wide analysis]), and by the electronic social media as well (Ahmed et al. 2020).

In first instance, the design of e-cigarettes as electronic nicotine delivery systems has focused the interest of toxicologists on nicotine and on local effects on the respiratory system, but other endpoints of toxicity are also being discussed (Tzortzi et al. 2020); current examples are atherosclerosis (Knura et al. 2018) and neuroinflammation (Heldt et al. 2020). The presence of a considerable number of toxic substances apart from nicotine in the liquid cartridges and in emissions of e-cigarettes has increasingly raised concern (Rehan et al. 2018).

With regard to possible carcinogenicity, a systematic literature search has just appeared by Bjurlin et al. (2020), using the preferred reporting items for systematic reviews and meta-analyses (PRISMA) guidelines. Relevant articles in peer-reviewed journals, published through January 2019 that investigated urinary biomarkers in e-cigarettes users were included. Parent compounds and urinary biomarkers were classified according to the *International Agency for Research on Cancer Monographs on the Evaluation of Carcinogenic Risks to Humans* and cross-referenced using the *Collaborative on Health and the Environment, Toxicant and Disease Database* to determine a link to bladder cancer, grouped by strength of evidence. The search identified 22 articles that met the final inclusion criteria and were included in the analysis. It finally appeared that, compared with non-user controls, e-cigarette users showed higher concentrations of urinary biomarkers of several carcinogenic compounds linked to bladder cancer.

This issue of *Archives of Toxicology* contains six contributions, which highlight the e-cigarette topic from very different angles: analytical chemistry, experimental toxicology, epidemiology and clinical aspects.

1. Mallock et al. (2020) performed a chemical characterization of liquids and aerosols of American JUUL pod e-cigarettes. To comply with European law (*Article 20 of the Tobacco Products Directive, 2014/40/EU*), the nicotine concentration in the liquids of the European JUUL pod version is limited to below 20 mg/ml. The vapour generation and nicotine delivery of the initial European version, a modified European version, and the original American high-nicotine JUUL pod variant were studied using a machine vaping set-up. Whereas the initial European version did not compensate for the lower nicotine content in the liquid, there was increased vaporization by the modified European version. As a consequence, nicotine delivery per puff approximated the American original. Mallock et al. (2020) concluded that there was similar addictiveness of both the enhanced European version and the original American product.
2. Following earlier subchronic inhalation studies in rats on the main e-cigarette components propylene glycol, glycerin and nicotine (Phillips et al. 2017), subchronic inhalation toxicity studies of aerosols from flavoured e-liquids are being reported by Ho et al. (2020). The results indicate that the inhalation of an e-liquid containing the neat mixture of “flavour group representatives” caused minimal local and systemic toxic effects. The biological effects related to exposure to the mixture with “flavour group representatives” were mainly nicotine-mediated, including changes in haematological and blood chemistry parameters, as well as organ weights. These results were interpreted to indicate no significant additive biological changes following inhalation exposure to the nebulized “flavour group representatives” mixture above the nicotine effects.
3. Chen et al. (2020) studied effects of e-cigarette vapour extract on phenotype and function of human monocyte-derived dendritic cells in vitro. The overall expression of 29 signaling molecules and other cytoplasmic proteins, mainly associated with DC activation, was significantly upregulated. The authors conclude that e-cigarette vapour moderately affects human dendritic cells, with effects are less pronounced than those of tobacco smoke.
4. Reumann et al. (2020) studied effects of e-vapour aerosols and cigarette smoke on bone morphology, structure, and strength in a 6-month inhalation study. Young ApoE^−/−^ mice were exposed to aerosols from three different e-vapour formulations. In view of a bone-preserving effect of e-vapour aerosols relative to cigarette smoke exposure, it was concluded that e-vapour products could potentially constitute less harmful alternatives to cigarettes in situations in which bone health is of importance.
5. Vitamin E acetate as a constituent of vaping liquids has come under scrutiny due to an association with e-cigarette (vaping) product use-associated lung injury (EVALI). There may be multiple causes for EVALI as a clinical syndrome. Feldman et al. (2020) applied the Bradford-Hill causation criteria to vitamin E acetate and the EVALI outbreak to clarify what further areas of study are needed to strengthen the causal argument. They highlight the need for systematized approaches to identify the cause of mass poisoning events of unknown etiology.
6. In a Guest Editorial, Javelle (2020) makes reference to recent clinical evidence (Chaumont et al. 2019) of pulmonary toxicity caused by vaping. It is suggested that this effect could interfere with disease symptoms of the respiratory system caused by the recent COVID-19 epidemic.

Viewing these different aspects together, it becomes very clear that a multidisciplinary approach is required to solve the complex questions that are associated with assessment and management of risks of e-cigarettes/vaping products. Explicitly, such contributions are invited for submission to Archives of Toxicology.
